# Supplementary material for: Identification of photoperiod-induced specific miRNAs in the adrenal glands of Sunite sheep (Ovis aries)
Source: Front Vet Sci. 2022 Jul 22;9:888207. doi: 10.3389/fvets.2022.888207 (PMC9354845; doi:10.3389/fvets.2022.888207)
Supplement: Supplementary file 1 [file Data_Sheet_1.ZIP › Supplementary file/miRDeep2 file.zip!/miRDeep2 file/Know_Structure/LP42A2/oar-mir-412.pdf]

miRBase precursor : oar-mir-412
Total read count : 478
oar-miR-412-3p read count : 458
oar-miR-412-5p read count : 10
remaining reads : 10

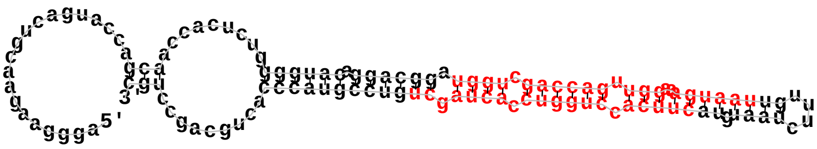

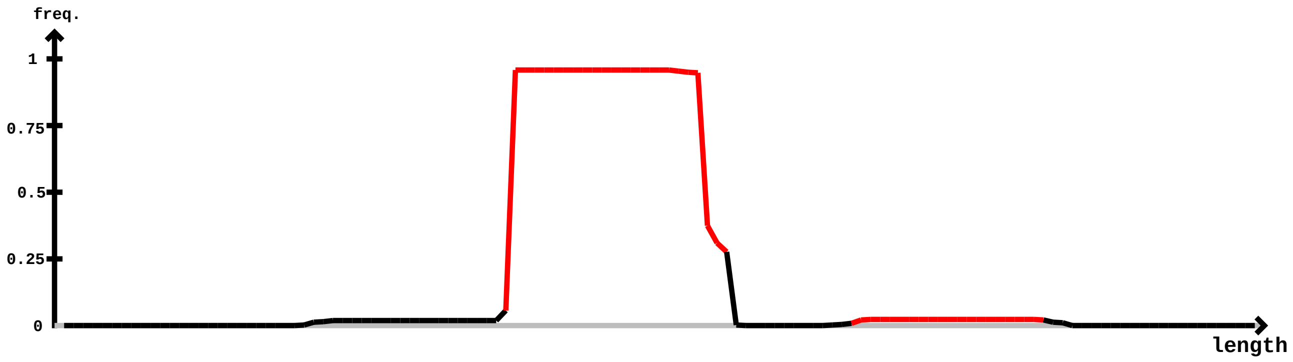


```
                                                                                  oar-miR-412-5p
                                              oar-miR-412-3p
5'- agggaagaacgucaguaccagcaaccacucugggguacaggacggauggucgaccaguuggaaaguaauuguuucuaauguacuucaccugguccacuagcuguccguacccacugcagccugc   -3'   exp
    ..........(.((((..(((........)))((((((.((((((.((((.((((((.(((..((((((((....)))).))))))).)))))).)))).)))))))))))))))))).......         reads   mm
    ........................ccacucugggguacaggacgga..............................................................................         1       0
    .........................cacucugggguacaggacgga..............................................................................         5       0
    ..........................acucugggguacaggacgga..............................................................................         1       0
    ...........................cucugggguacaggacgg...............................................................................         1       0
    ...........................cucugggguacaggacgga..............................................................................         1       0
    .............................................auggucgaccaguuggaaa............................................................         1       0
    .............................................auggucgaccaguuggaaagu..........................................................         16      0
    .............................................auggucgaccaguuggaaagua.........................................................         2       0
    ..............................................uggucgaccaguuggaa.............................................................         2       0
    ..............................................uggucgaccaguuggaaa............................................................         1       0
    ..............................................uggucgaccaguuggaaag...........................................................         1       0
    ..............................................uggucgaccaguuggaaagu..........................................................         258     0
    ..............................................uggucgaccaguuggaaagua.........................................................         29      0
    ..............................................uggucgaccaguuggaaaguaa........................................................         16      0
    ..............................................uggucgaccaguuggaaaguaau.......................................................         131     0
    ..............................................uggucgaccaguuggaaaguaauu......................................................         1       0
    ...............................................................................guacuucaccugguccacuagc.......................         1       0
    ................................................................................uacuucaccugguccacuagcugu....................         1       0
    .................................................................................acuucaccugguccacuagcu......................         2       0
    ..................................................................................cuucaccugguccacuagcu......................         1       0
    ..................................................................................cuucaccugguccacuagcug.....................         1       0
    ..................................................................................cuucaccugguccacuagcugu....................         4       0
    ...................................................................................uucaccugguccacuagcu......................         1       0
```
